# Bis[glycinium(0.5+)] perrhenate

**DOI:** 10.1107/S160053680803849X

**Published:** 2008-12-06

**Authors:** V. H. Rodrigues, M. M. R. R. Costa, T. Dekola, E. de Matos Gomes

**Affiliations:** a CEMDRX, Department of Physics, University of Coimbra, P-3004-516 Coimbra, Portugal; b Departamento de Física, Universidade do Minho, P-4710-057 Braga, Portugal

## Abstract

All the residues of the title compound, (C_2_H_5.5_NO_2_)_2_[ReO_4_], are located in general crystallographic positions. The glycine mol­ecules have usual conformations [Rodrigues Matos Beja *et al.* (2006 ▶). *Acta Cryst*. C**62**, o71–o72] with the H atom of the carboxylate group half-occupied, thus bearing a formal half-positive charge per molecule. The perrhenate anion has nearly ideal tetra­hedral geometry. A large number of strong hydrogen bonds give rise to the overall three-dimensional network. A two-dimensional network, parallel to (100), is made up of strong O—H⋯O hydrogen bonds with a donor acceptor distance of 2.445 (2) Å. A large number of weaker O—H⋯O and N—H⋯O hydrogen bonds consolidates the structure into an overall three-dimensional network.

## Related literature

For a related structure, see: Rodrigues *et al.* (2006 ▶).
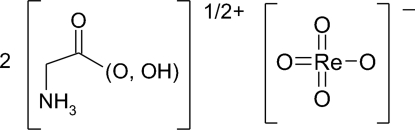


## Experimental

### 

#### Crystal data

(C_2_H_5.5_NO_2_)_2_[ReO_4_]
*M* *_r_* = 401.35
Monoclinic, 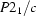

*a* = 15.7095 (5) Å
*b* = 8.1826 (3) Å
*c* = 8.2909 (3) Å
β = 103.7152 (16)°
*V* = 1035.36 (6) Å^3^
*Z* = 4
Mo *K*α radiation
μ = 11.77 mm^−1^
*T* = 291 (2) K
0.15 × 0.13 × 0.10 mm

#### Data collection

Bruker APEXII diffractometer
Absorption correction: multi-scan (*SADABS*; Sheldrick, 2003 ▶) *T*_min_ = 0.18, *T*_max_ = 0.31
78826 measured reflections
8587 independent reflections
6232 reflections with *I* > 2σ(*I*)
*R*_int_ = 0.038

#### Refinement

*R*[*F* ^2^ > 2σ(*F* ^2^)] = 0.030
*wR*(*F* ^2^) = 0.055
*S* = 1.06
8587 reflections
141 parameters
H-atom parameters constrained
Δρ_max_ = 2.33 e Å^−3^
Δρ_min_ = −2.87 e Å^−3^

### 

Data collection: *APEX2* (Bruker–Nonius, 2004 ▶); cell refinement: *SAINT* (Bruker, 2003 ▶); data reduction: *SAINT*; program(s) used to solve structure: *SHELXS97* (Sheldrick, 2008 ▶); program(s) used to refine structure: *SHELXL97* (Sheldrick, 2008 ▶); molecular graphics: *ORTEPII* (Johnson, 1976 ▶); software used to prepare material for publication: *SHELXL97* and *PLATON* (Spek, 2003 ▶).

## Supplementary Material

Crystal structure: contains datablocks I, global. DOI: 10.1107/S160053680803849X/rn2049sup1.cif

Structure factors: contains datablocks I. DOI: 10.1107/S160053680803849X/rn2049Isup2.hkl

Additional supplementary materials:  crystallographic information; 3D view; checkCIF report

## Figures and Tables

**Table 1:** Hydrogen-bond geometry (Å, °)

*D*—H⋯*A*	*D*—H	H⋯*A*	*D*⋯*A*	*D*—H⋯*A*
O12—H12⋯O22^i^	0.82	1.64	2.445 (2)	167
N11—H11*A*⋯O11^ii^	0.89	2.02	2.869 (2)	158
N11—H11*B*⋯O21	0.89	2.18	3.003 (2)	153
N11—H11*B*⋯O2	0.89	2.47	3.000 (3)	119
N11—H11*C*⋯O11^iii^	0.89	1.94	2.830 (3)	175
O22—H22⋯O12^i^	0.82	1.64	2.445 (2)	165
N21—H21*A*⋯O21	0.89	2.27	2.738 (3)	113
N21—H21*A*⋯O1	0.89	2.29	3.136 (4)	158
N21—H21*B*⋯O3^iv^	0.89	2.10	2.896 (3)	149
N21—H21*B*⋯O1^v^	0.89	2.60	3.274 (4)	133
N21—H21*C*⋯O4^vi^	0.89	2.14	2.794 (3)	130
N21—H21*C*⋯O1^vii^	0.89	2.37	3.012 (3)	130

Symmetry codes: (i) 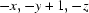; (ii) 
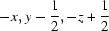
; (iii) 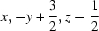; (iv) 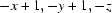; (v) 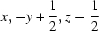; (vi) 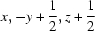; (vii) 
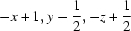
.

**Fig. 1.**
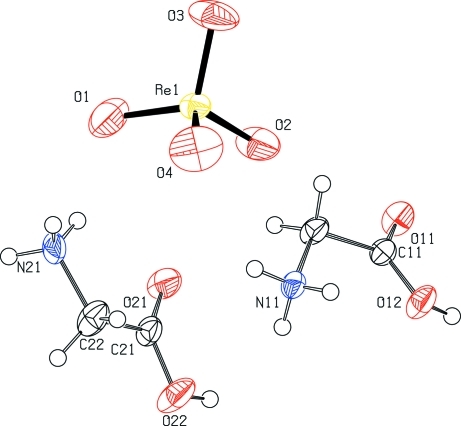
ORTEPII (Johnson, 1976) plot of the title compound. Displacement ellipsoids are drawn at the 50% level.

**Fig. 2.**
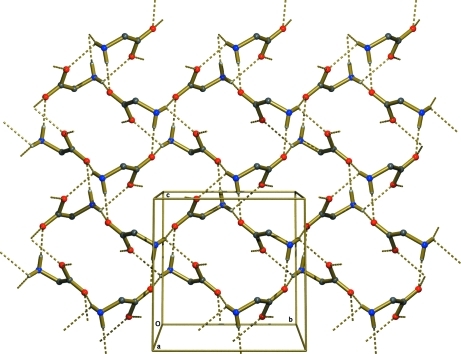
Packing of glycine molecules showing the (100) network.

## supplementary crystallographic information

### Experimental

Crystals of the monoclinic polymorph of glycinium glycine perrhenate were obtained from a water solution of analytical grade reagents glycine(99.5%) and perrhenic acid solution (65–75% water, 99.5%), purchased from Aldrich, in a 2:1 molar ratio.

### Refinement

The structure was solved by direct methods using *SHELXS97*. All H atoms were first located on a difference Fourier map; those bonded to C atoms and carboxyl O atoms were placed at idealized positions and refined as riding [C—H=0.97 and 0.98 Å, O—H=0.82 Å, *U*_iso_(H)=1.2*U*_eq_(C) and *U*_iso_(H)=1.5*U*_eq_(O)].

Examination of the crystal structure with *PLATON* (Spek, 2003) showed that there are no solvent-accessible voids in the crystal lattice.

### Crystal data

**Table:**

2(C_2_H_5.5_NO_2_)[ReO_4_]	*F*(000) = 752
*M**_r_* = 401.35	*D*_x_ = 2.575 Mg m^−^^3^
Monoclinic, *P*2_1_/*c*	Mo *K*α radiation, λ = 0.71073 Å
Hall symbol: -P 2ybc	Cell parameters from 7839 reflections
*a* = 15.7095 (5) Å	θ = 4.0–40.1°
*b* = 8.1826 (3) Å	µ = 11.77 mm^−^^1^
*c* = 8.2909 (3) Å	*T* = 291 K
β = 103.7152 (16)°	Block, translucent colourless
*V* = 1035.36 (6) Å^3^	0.15 × 0.13 × 0.10 mm
*Z* = 4	

### Data collection

**Table:**

Bruker APEXII diffractometer	8587 independent reflections
Radiation source: fine-focus sealed tube	6232 reflections with *I* > 2σ(*I*)
graphite	*R*_int_ = 0.038
φ and ω scans	θ_max_ = 45.3°, θ_min_ = 2.7°
Absorption correction: multi-scan (*SADABS*; Sheldrick, 2003)	*h* = −30→31
*T*_min_ = 0.18, *T*_max_ = 0.31	*k* = −16→15
78826 measured reflections	*l* = −16→16

### Refinement

**Table:**

Refinement on *F*^2^	Secondary atom site location: difference Fourier map
Least-squares matrix: full	Hydrogen site location: inferred from neighbouring sites
*R*[*F*^2^ > 2σ(*F*^2^)] = 0.030	H-atom parameters constrained
*wR*(*F*^2^) = 0.055	*w* = 1/[σ^2^(*F*_o_^2^) + (0.001*P*)^2^ + 2.2865*P*] where *P* = (*F*_o_^2^ + 2*F*_c_^2^)/3
*S* = 1.06	(Δ/σ)_max_ = 0.002
8587 reflections	Δρ_max_ = 2.33 e Å^−^^3^
141 parameters	Δρ_min_ = −2.87 e Å^−^^3^
0 restraints	Extinction correction: *SHELXL97* (Sheldrick, 2008), Fc^*^=kFc[1+0.001xFc^2^λ^3^/sin(2θ)]^-1/4^
Primary atom site location: structure-invariant direct methods	Extinction coefficient: 0.00614 (15)

### Special details

**Table:**

Geometry. All e.s.d.'s (except the e.s.d. in the dihedral angle between two l.s. planes) are estimated using the full covariance matrix. The cell e.s.d.'s are taken into account individually in the estimation of e.s.d.'s in distances, angles and torsion angles; correlations between e.s.d.'s in cell parameters are only used when they are defined by crystal symmetry. An approximate (isotropic) treatment of cell e.s.d.'s is used for estimating e.s.d.'s involving l.s. planes.
Refinement. Refinement of *F*^2^ against ALL reflections. The weighted *R*-factor *wR* and goodness of fit *S* are based on *F*^2^, conventional *R*-factors *R* are based on *F*, with *F* set to zero for negative *F*^2^. The threshold expression of *F*^2^ > σ(*F*^2^) is used only for calculating *R*-factors(gt) *etc*. and is not relevant to the choice of reflections for refinement. *R*-factors based on *F*^2^ are statistically about twice as large as those based on *F*, and *R*- factors based on ALL data will be even larger.

### Fractional atomic coordinates and isotropic or equivalent isotropic displacement parameters (Å^2^)

**Table:**

	*x*	*y*	*z*	*U*_iso_*/*U*_eq_	Occ. (<1)
Re1	0.384633 (5)	0.604894 (11)	0.053166 (13)	0.02896 (3)	
O1	0.43021 (16)	0.4923 (4)	0.2269 (3)	0.0636 (7)	
O2	0.28327 (14)	0.6733 (3)	0.0625 (4)	0.0633 (8)	
O3	0.44936 (16)	0.7699 (3)	0.0397 (5)	0.0685 (9)	
O4	0.3784 (2)	0.4775 (4)	−0.1121 (3)	0.0698 (8)	
O11	0.02851 (12)	0.9119 (2)	0.2663 (2)	0.0343 (4)	
O12	−0.02823 (11)	0.7477 (2)	0.0538 (2)	0.0366 (4)	
H12	−0.0747	0.7890	0.0614	0.055*	0.50
C11	0.03285 (13)	0.7965 (3)	0.1730 (3)	0.0242 (3)	
C12	0.11921 (13)	0.7067 (3)	0.1971 (3)	0.0291 (4)	
H12A	0.1608	0.7754	0.1588	0.035*	
H12B	0.1424	0.6864	0.3145	0.035*	
N11	0.11042 (11)	0.5501 (2)	0.1070 (2)	0.0253 (3)	
H11A	0.0761	0.4833	0.1479	0.038*	
H11B	0.1631	0.5048	0.1189	0.038*	
H11C	0.0868	0.5676	−0.0002	0.038*	
O21	0.24920 (12)	0.3008 (2)	0.0961 (3)	0.0409 (4)	
O22	0.16419 (12)	0.1024 (3)	−0.0423 (3)	0.0504 (6)	
H22	0.1241	0.1641	−0.0353	0.076*	0.50
C21	0.23754 (14)	0.1710 (3)	0.0210 (3)	0.0281 (4)	
C22	0.31325 (16)	0.0716 (3)	−0.0074 (4)	0.0373 (5)	
H22A	0.3076	−0.0399	0.0286	0.045*	
H22B	0.3109	0.0690	−0.1254	0.045*	
N21	0.39933 (12)	0.1372 (3)	0.0823 (3)	0.0337 (4)	
H21A	0.3923	0.2372	0.1194	0.051*	
H21B	0.4351	0.1417	0.0136	0.051*	
H21C	0.4224	0.0724	0.1676	0.051*	

### Atomic displacement parameters (Å^2^)

**Table:**

	*U*^11^	*U*^22^	*U*^33^	*U*^12^	*U*^13^	*U*^23^
Re1	0.02220 (4)	0.02717 (4)	0.04083 (5)	−0.00013 (3)	0.01407 (3)	−0.00404 (3)
O1	0.0401 (12)	0.093 (2)	0.0524 (14)	−0.0127 (13)	−0.0001 (10)	0.0177 (14)
O2	0.0333 (10)	0.0379 (10)	0.130 (2)	0.0040 (8)	0.0416 (13)	−0.0007 (13)
O3	0.0467 (12)	0.0383 (11)	0.136 (3)	−0.0090 (9)	0.0521 (16)	−0.0019 (14)
O4	0.080 (2)	0.083 (2)	0.0523 (15)	−0.0094 (16)	0.0276 (14)	−0.0268 (14)
O11	0.0309 (7)	0.0398 (9)	0.0320 (8)	0.0054 (7)	0.0069 (6)	−0.0106 (7)
O12	0.0226 (7)	0.0378 (9)	0.0440 (10)	0.0077 (6)	−0.0031 (7)	−0.0144 (7)
C11	0.0206 (7)	0.0270 (8)	0.0257 (9)	0.0011 (6)	0.0068 (6)	−0.0006 (7)
C12	0.0194 (7)	0.0314 (9)	0.0348 (11)	0.0015 (7)	0.0029 (7)	−0.0069 (8)
N11	0.0187 (6)	0.0262 (7)	0.0300 (9)	0.0035 (5)	0.0043 (6)	0.0002 (6)
O21	0.0259 (7)	0.0310 (8)	0.0635 (13)	0.0041 (6)	0.0063 (8)	−0.0144 (8)
O22	0.0219 (7)	0.0475 (11)	0.0747 (15)	0.0057 (7)	−0.0029 (8)	−0.0284 (11)
C21	0.0213 (8)	0.0288 (9)	0.0323 (10)	0.0054 (7)	0.0027 (7)	−0.0034 (8)
C22	0.0254 (9)	0.0429 (13)	0.0417 (13)	0.0092 (9)	0.0043 (9)	−0.0133 (10)
N21	0.0217 (7)	0.0309 (9)	0.0490 (12)	0.0096 (6)	0.0094 (8)	0.0134 (8)

### Geometric parameters (Å, °)

**Table:**

Re1—O4	1.706 (3)	N11—H11B	0.8900
Re1—O2	1.707 (2)	N11—H11C	0.8900
Re1—O3	1.710 (2)	O21—C21	1.223 (3)
Re1—O1	1.717 (3)	O22—C21	1.277 (3)
O11—C11	1.233 (3)	O22—H22	0.8200
O12—C11	1.267 (3)	C21—C22	1.505 (3)
O12—H12	0.8200	C22—N21	1.481 (3)
C11—C12	1.514 (3)	C22—H22A	0.9700
C12—N11	1.473 (3)	C22—H22B	0.9700
C12—H12A	0.9700	N21—H21A	0.8900
C12—H12B	0.9700	N21—H21B	0.8900
N11—H11A	0.8900	N21—H21C	0.8900
			
O4—Re1—O2	111.10 (15)	C12—N11—H11C	109.5
O4—Re1—O3	110.60 (15)	H11A—N11—H11C	109.5
O2—Re1—O3	108.64 (11)	H11B—N11—H11C	109.5
O4—Re1—O1	106.21 (17)	C21—O22—H22	109.5
O2—Re1—O1	110.24 (14)	O21—C21—O22	127.1 (2)
O3—Re1—O1	110.03 (15)	O21—C21—C22	121.4 (2)
C11—O12—H12	109.5	O22—C21—C22	111.5 (2)
O11—C11—O12	125.87 (19)	N21—C22—C21	112.7 (2)
O11—C11—C12	118.03 (19)	N21—C22—H22A	109.0
O12—C11—C12	116.08 (18)	C21—C22—H22A	109.0
N11—C12—C11	112.44 (17)	N21—C22—H22B	109.0
N11—C12—H12A	109.1	C21—C22—H22B	109.0
C11—C12—H12A	109.1	H22A—C22—H22B	107.8
N11—C12—H12B	109.1	C22—N21—H21A	109.5
C11—C12—H12B	109.1	C22—N21—H21B	109.5
H12A—C12—H12B	107.8	H21A—N21—H21B	109.5
C12—N11—H11A	109.5	C22—N21—H21C	109.5
C12—N11—H11B	109.5	H21A—N21—H21C	109.5
H11A—N11—H11B	109.5	H21B—N21—H21C	109.5
			
O11—C11—C12—N11	166.3 (2)	O21—C21—C22—N21	7.3 (4)
O12—C11—C12—N11	−15.7 (3)	O22—C21—C22—N21	−172.9 (2)

### Hydrogen-bond geometry (Å, °)

**Table:**

*D*—H···*A*	*D*—H	H···*A*	*D*···*A*	*D*—H···*A*
O12—H12···O22^i^	0.82	1.64	2.445 (2)	167
N11—H11A···O11^ii^	0.89	2.02	2.869 (2)	158
N11—H11B···O21	0.89	2.18	3.003 (2)	153
N11—H11B···O2	0.89	2.47	3.000 (3)	119
N11—H11C···O11^iii^	0.89	1.94	2.830 (3)	175
O22—H22···O12^i^	0.82	1.64	2.445 (2)	165
N21—H21A···O21	0.89	2.27	2.738 (3)	113
N21—H21A···O1	0.89	2.29	3.136 (4)	158
N21—H21B···O3^iv^	0.89	2.10	2.896 (3)	149
N21—H21B···O1^v^	0.89	2.60	3.274 (4)	133
N21—H21C···O4^vi^	0.89	2.14	2.794 (3)	130
N21—H21C···O1^vii^	0.89	2.37	3.012 (3)	130

Symmetry codes: (i) −*x*, −*y*+1, −*z*; (ii) −*x*, *y*−1/2, −*z*+1/2; (iii) *x*, −*y*+3/2, *z*−1/2; (iv) −*x*+1, −*y*+1, −*z*; (v) *x*, −*y*+1/2, *z*−1/2; (vi) *x*, −*y*+1/2, *z*+1/2; (vii) −*x*+1, *y*−1/2, −*z*+1/2.

## References

[bb1] Bruker (2003). *SAINT* Bruker AXS Inc., Madison, Wisconsin, USA.

[bb2] Bruker (2004). *APEX2* Bruker AXS Inc., Madison, Wisconsin, USA.

[bb3] Johnson, C. K. (1976). *ORTEPII* Report ORNL-5138. Oak Ridge National Laboratory, Tennessee, USA.

[bb4] Rodrigues, V. H., Matos Beja, A., Paixão, J. A. & Costa, M. M. R. R. (2006). *Acta Cryst.* C**62**, o71–o72. 10.1107/S0108270105040953 16456289

[bb5] Sheldrick, G. M. (2003). *SADABS* Bruker AXS Inc., Madison, Wisconsin, USA.

[bb6] Sheldrick, G. M. (2008). *Acta Cryst.* A**64**, 112–122. 10.1107/S0108767307043930 18156677

[bb7] Spek, A. L. (2003). *J. Appl. Cryst.* **36**, 7–13.

